# Effects of an educational health promotion intervention to improve human papillomavirus vaccination acceptance and uptake among adolescent girls: a cluster randomized controlled trial

**DOI:** 10.1186/s12889-025-24511-4

**Published:** 2025-10-09

**Authors:** Suzanne Hoi Shan Lo, Janita Pak Chun Chau, Kai Chow Choi, Laveeza Butt, Grace Chung Yan Lui, Vivian Wing Yan Lee, Alexander Yuk Lun Lau

**Affiliations:** 1 The Nethersole School of Nursing, The Chinese University of Hong Kong, Hong Kong SAR, China; 2 Department of Medicine and Therapeutics, Faculty of Medicine, The Chinese University of Hong Kong, Hong Kong SAR, China; 3 Centre for Learning Enhancement And Research, The Chinese University of Hong Kong, Hong Kong SAR, China

**Keywords:** Adolescents, Attitudes, Human papillomavirus, Human papillomavirus vaccination, Immunization, Knowledge, Randomized controlled trial

## Abstract

**Background:**

Human Papillomavirus (HPV) is the most common sexually-transmitted infection worldwide and the primary cause of cervical cancer. Although timely vaccination can prevent HPV infection and reduce the risk of cervical cancer, vaccine uptake among target groups such as adolescent girls is suboptimal. This study aimed to investigate the effects of a school-based educational health promotion intervention to improve HPV vaccine acceptance and uptake among young adolescent girls.

**Methods:**

A cluster randomized controlled trial was conducted with 1,340 female adolescents (Mean age: 15.3 years [S.D.: 1.1]) recruited from 18 secondary schools. Recruited schools and the respective participants were randomized to either the intervention or control group in a 1:1 ratio. The school-based intervention was delivered by a registered nurse and included a health talk, small group discussions, and an online game covering topics such as stages of cervical cancer, HPV infection, and HPV vaccination. Outcome measures were participants’ uptake of HPV vaccination, intention to receive HPV vaccination, and knowledge and attitudes towards HPV vaccination, which were collected at either or both 1-month and 1-year post-intervention. Data were analyzed through a random intercept mixed-effects model.

**Results:**

No significant differences were found in the HPV vaccine uptake rate and uptake intention of the two groups, though more participants in the intervention group (11.9%) were vaccinated at 1-year post-intervention compared to those in the control group (7.9%). Significant improvements were found in the intervention group’s attitudes towards HPV vaccination, particularly in the subscales of Barriers (B=-2.20, 95% CI, [-3.46, -0.94], *p* = 0.001), Risk denial (B=-0.82, 95% CI, [-1.60, -0.05], *p* = 0.038), and Uncertainty (B = 0.72, 95% CI, [0.16, 1.27], *p* = 0.011). Significant improvements were also found in the intervention group’s knowledge of HPV (B = 0.94, 95% CI, [0.16, 1.72], *p* = 0.018).

**Conclusions:**

The programme was beneficial in improving adolescent girls’ knowledge and attitudes, though there is room for optimization in order to significantly improve vaccination intent and uptake. Future studies may investigate lengthier interventions involving other stakeholders such as parents and healthcare providers. An adapted programme may also be investigated among other demographics such as older females and adolescent boys.

**Trial registration:**

ClinicalTrials.gov, NCT04438291; Registration date: 2020-06-18.

**Supplementary Information:**

The online version contains supplementary material available at 10.1186/s12889-025-24511-4.

## Background

Cervical cancer is the fourth most common cancer in women globally and the seventh most common cancer among women in Hong Kong [1, 2]. It is caused by persistent infection with human papillomavirus (HPV), which is the most common sexually-transmitted infection worldwide and has over 100 identified strains causing various health issues ranging from warts to anogenital cancers. HPV vaccination is an effective form of prevention against infection with prevalent cancer-causing HPV subtypes such as HPV-16 and 18, which are responsible for the vast majority of cervical cancer cases [3]. In recent years, studies investigating the effects of large-scale national HPV immunization campaigns have reported reduced HPV infections among vaccinated populations and associated declines of up to 80% in the rate of cervical cancer incidence [4, 5]. Despite these encouraging findings, the acceptance and uptake of HPV vaccination remains suboptimal in different regions, with final dose uptake by girls aged 9–15 years reaching 35% in Europe and North America, and only 4% in East and South East Asia [6], thus hindering the elimination of cervical cancer as a major global public health concern.

As the protection offered by HPV vaccination is strongest when administered before initial exposure to the virus, the primary target group for vaccination is girls aged 9–14 years, followed by older girls, women, and boys as secondary targets. However, vaccine hesitancy is a major barrier affecting HPV vaccine uptake, with poor understanding and views towards the vaccination causing key stakeholders such as parents of adolescents to refuse immunization [7]. Common concerns affecting the decision to arrange HPV vaccination for their children include insufficient information about vaccination risks, poor interactions with healthcare workers, and misconceptions about the HPV vaccination [8, 9]. Similarly, adolescents themselves also demonstrate hesitation regarding HPV vaccination due to doubts regarding long-term effectiveness, their level of infection risk, and fear of potential side effects [10]. To improve vaccine uptake, it is thus necessary to provide stakeholders with sufficient health information from reliable sources in order to mitigate their concerns and increase the likelihood of receiving timely HPV vaccination.

There is evidence that educational health promotion interventions could reduce vaccine hesitancy and have positive effects on HPV vaccine uptake by addressing knowledge gaps and improving attitudes [11]. A study of 434 female students in Greece showed that an interactive informational school-based intervention significantly increased both students’ knowledge of HPV and willingness to receive the vaccination [12]. Similarly, a systematic review of 16 studies supports the use of educational interventions to increase vaccination intention as well as uptake [13]. In contrast, some studies present mixed findings on the effects of educational interventions on vaccine uptake rate, suggesting that education alone may be insufficient to prompt HPV vaccine initiation and that additional components such as involving vaccine providers may have a stronger effect on raising vaccine uptake [14]. More evidence on optimal formats of HPV vaccine promotion interventions is thus required.

In recent years, Hong Kong’s Department of Health has started to provide free HPV vaccination for female students in Grades 5–6 (ages 10–12 years) as part of the Childhood Immunization Programme, thus initiating large-scale HPV vaccine coverage for a key target group. As of writing, up to 90% of eligible girls have received two doses of the vaccination, signaling a strong uptake rate and significant step towards herd immunity [15]. However, older adolescent and young adult females are not covered by the programme and have to arrange for the vaccination themselves, leading to a lower overall vaccine coverage rate among young at-risk females. Moreover, a previous study found that HPV vaccine uptake among secondary school girls in Hong Kong was lower than 10%, and suggested that poor knowledge and lack of awareness may be reasons behind low uptake [16]. In order to address informational needs and support the decision-making process regarding HPV vaccination in this demographic, we developed a multidisciplinary team-led school-based HPV vaccination health promotion programme (MDL-SHPVP) for female adolescents and their parents/guardians. This study was conducted to explore the effects of the programme on female adolescents’ vaccination intention, knowledge, attitudes, and uptake of HPV vaccination. Findings are expected to provide preliminary information for the promotion of HPV vaccination in school-based settings for female adolescents, which can be adapted for use among secondary target groups such as adolescent boys and older females.

## Methods

### Design and settings

This was a cluster randomized controlled trial (CRT) aiming to explore the effects of the MDL-SHPVP programme on female adolescents’ knowledge, attitudes, and uptake of HPV vaccination. Female students between the ages of 12–18 years who had not received HPV vaccination were included in the study. Participants were recruited from secondary schools in Hong Kong from 2021 to 2023. Inclusion criteria were as follows: (1) female aged between 12 and 18 years old; (2) has not received HPV vaccination.

Those who had (1) received HPV vaccination, (2) a history of severe life-threatening allergies to a previous dose of HPV vaccine or vaccine component, (3) a history of immediate hypersensitivity to yeast, (4) moderate or severe acute illness, and (5) participated in previous trials related to HPV vaccination were excluded from the study.

### Sample size

The sample size was estimated based on the primary outcome of the HPV vaccine uptake rate at 1-year post-intervention. Several studies revealed the low overall uptake rate of the HPV vaccine (< 10%) in Hong Kong [16–18]. We anticipated that the MDL-SHPVP could double the uptake rate and therefore set this target for sample size estimation a priori. We used the power analysis software PASS 16 (NCSS, Kaysville, UT) to estimate that a sample size of 266 participants in the intervention and control groups would be required to achieve 90% power at a 2-sided 5% level of significance to detect a net difference of at least 10% in the uptake rate between the 2 groups, assuming that the rate would be 10% for the control group. To allow for an attrition rate of up to 20%, a total of 666 participants with 333 in each group would be needed. Furthermore, to account for clustering design with an intracluster correlation coefficient of up to 0.02 [19, 20], a total of 18 schools with at least 140 female adolescents from each school were required.

### Randomization

Cluster randomization was implemented at the school level to avoid potential contamination between the intervention and control groups. Recruited schools were randomized in a 1:1 ratio to either the intervention or control group, with all eligible participants recruited from the same school allocated to either group. An independent statistician used computer-based randomization to generate a random sequence of group identifiers, according to which group allocation was carried out based on the sequence of enrollment of the participating schools. The allocations were kept concealed from outcome assessors and investigators.

### Intervention

The MDL-SHPVP was developed for this study based on the tenets of the Health Belief Model and delivered by a volunteer team led by a registered nurse [21]. The programme comprised a health talk, small group discussions, and an educational online game. The health talk and group discussions were held on school premises or online on a video conferencing platform depending on social distancing guidelines at the time of intervention delivery. A link to the online game was provided to the participants and teacher-in-charge for circulation. The programme was conducted by a nurse-led team of lay volunteers and healthcare professionals. Accordingly, multi-session training on intervention delivery was provided to a total of 128 volunteers (mostly undergraduate students) and 30 healthcare professionals.

Several types of educational content were developed for the programme, including six video interviews with healthcare professionals, three sharing videos of vaccinated adolescents and their parents, and an information booklet, all of which were tested during a prior user engagement study and found to be informative and appropriate [22]. Topics covered in the materials included an introduction to HPV infection, methods of transmission and infection risk, cervical cancer, available HPV vaccines, the benefits and risks of immunization, common myths and misconceptions about HPV and the vaccination, experiences of receiving HPV vaccination and the decision-making process, and information on where to receive vaccination in Hong Kong. The intervention lasted between 2 and 2.5 h depending on participant engagement during question and discussion sections.

Participants allocated to the control group were provided with a 30-minute educational video about HPV vaccination which covered the topics of HPV infection, cervical cancer, and HPV vaccination to be viewed in their own time.

### Outcome measures

The primary outcome is female adolescents’ uptake rate of the HPV vaccine 1-year post-intervention. Secondary outcomes include uptake rate of the HPV vaccine 1-month post-intervention and changes in female adolescents’ intention to receive HPV vaccination, HPV knowledge, and attitudes and beliefs (from baseline to 1-month post-intervention). Data were collected through a combination of physical and digital questionnaires at baseline, 1-month, and 1-year post-intervention. Considering that the decision to receive HPV vaccination is usually dependent on adolescents’ parents, the two follow-up points at 1-month and 1-year were deemed to allow for adequate time for discussions among stakeholders and making relevant arrangements for receiving HPV vaccination. Physical questionnaires were distributed to the teachers-in-charge of each school who would assist in following up with data collection. Digital questionnaires were distributed directly to participants by the research team as well as teachers-in-charge.

#### HPV vaccination status

Information about adolescents’ HPV vaccination status was collected twice through a self-report questionnaire at 1-month and 1-year post-intervention.

#### HPV vaccination intention

Adolescents’ intention to receive HPV vaccination was evaluated on a 10-point Likert scale (1 = definitely not, 10 = definitely) [23] and reasons for no or low intention (a score of < 5) were collected.

#### Attitudes and beliefs regarding HPV vaccination

Adolescents’ attitudes and beliefs about HPV vaccination were measured by the 17-item modified Carolina HPV Immunization Attitudes and Beliefs Scale (CHIAS) [24]. Each item is rated on an 11-point scale, with higher values indicating lower approval of HPV vaccination. The Chinese version of the scale (CHIAS-C) was used in this study. The translated scale has been validated in a population of Chinese female adolescents and found to be a reliable tool with acceptable internal consistency and convergent validity [25]. 

#### Knowledge about HPV and HPV vaccine

A 23-item HPV knowledge scale (GK23) and a 9-item vaccination knowledge scale (VK9) were used to measure adolescents’ knowledge about HPV and the HPV vaccine [26]. Items are rated either “true,” “false,” or “don’t know”, with a higher score reflecting better knowledge. The Chinese language versions of the GK23 and VK9 were used in this study. Both scales have been validated and show acceptable internal consistency, with Kuder-Richardson 20 scores of 0.88 for the GK23 and 0.75 for the VK9 [27]. 

#### Socio-demographic and vaccination information

The following socio-demographic and vaccination information was collected: adolescents’ age and immunization history; receipt of influenza and HPV vaccine; participation in educational talks on HPV vaccination; HPV immunization of family members; and whether vaccinations have been discussed with healthcare professionals.

### Statistical analysis

Data were summarized and presented using appropriate descriptive statistics. Normality of continuous variables was assessed based on their skewness and kurtosis statistics and normal probability plots. No continuous variable was found deviated from normality. Considering the potential design effect of the randomisation conducted at the school level instead of by individual participant, the outcome analyses were performed on the basis of a mixed-effects model with up to three levels to account for inter-correlation among individuals within the same school and for intra-correlation over time within an individual and variations between individuals. More specifically, a (two-level) random intercept mixed-effects model was used to compare the HPV uptake rate at T1 and T2 between the intervention and control groups accounting for the clustering design, while a (three-level) random intercept mixed-effects model was used to compare the differential change for each of the repeated measures outcomes at T1 with respect to T0 accounting for the intra-correlated clustered and repeated-measures data. The restricted maximum likelihood method was used in the mixed-effects models to handle the missing data which could produce unbiased estimates even in the presence of missing data, provided that the data are missing at random. As the study was an exploratory trial which was not to confirm the effectiveness of the intervention, multiplicity adjustment was therefore not made. Odds ratio and Cohen’s d together with their 95% confidence intervals were respectively calculated for the vaccine uptake and other continuous outcomes based on their change score at T1 with respect to T0 to quantify the effect sizes of the intervention. Cohen’s d effect sizes of 0.2, 0.5 and 0.8 are conventionally interpreted as small, medium and large, respectively [28]. All statistical analyses were conducted using SAS release 9.4 (SAS Institute Inc, Cary, NC) with PROC MIXED and NLMIXED for the mixed-effects models. The level of significance was set at 0.05 (2-sided).

### Deviations from the protocol

The collection of student data was challenged by COVID-19 pandemic that caused suspension of classes, study from home, and packed study schedule limiting access to students to join the study. Therefore, the number of students recruited was lower than the estimated sample size. Moreover, the intervention was delivered in a mixed face-to-face and video conferencing format due to social distancing guidelines in force during the study.

### Ethical considerations

was obtained from the Joint Chinese University of Hong Kong-New Territories East Cluster Clinical Research Ethics Committee (Ref. no.: 2019.055). The rights and safety of participants were protected in accordance with local laws, the Declaration of Helsinki, institutional policies, and the ICH guideline for good clinical practice. Informed consent was received from adolescents and their parents. Participants were informed of their right to refuse participation or withdraw from the study at any point. All data were encrypted and stored securely with access available only to the research team members.

## Results

**Fig. 1:**
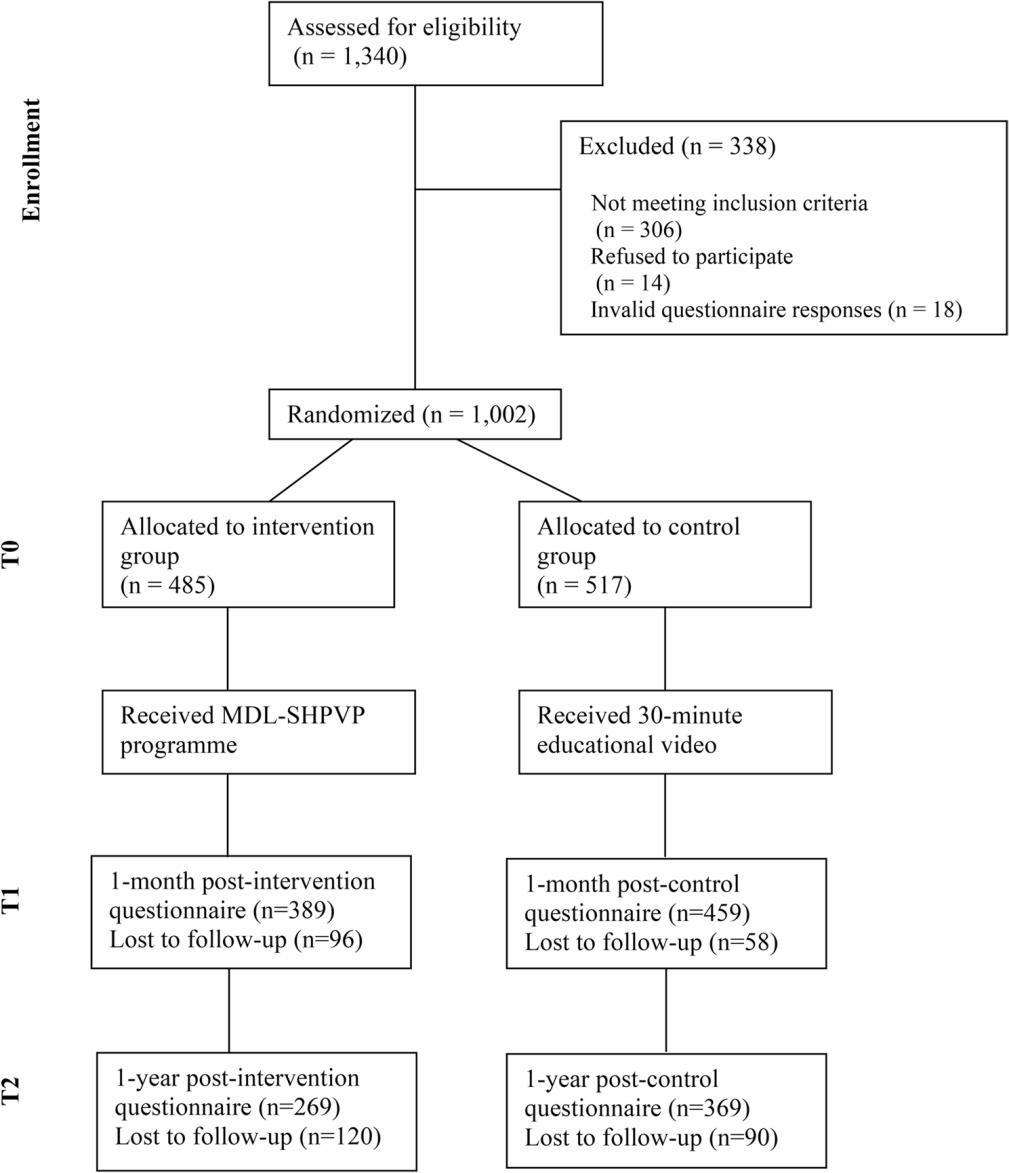
Flow of participants in the study

A total of 1,340 female students from 18 secondary schools were recruited. Of the 18 schools, 9 were randomly allocated to each of the intervention and control groups. Among the students, 338 students were excluded because they received HPV vaccination prior to enrolment (*n* = 306), provided invalid information on the questionnaires (*n* = 18), or declined to participate in the study (*n* = 14). Therefore, a total of 1,002 female students were included in the analysis. Out of these students, a total of 848 (84.6%) and 638 (63.7%) students completed the questionnaires at one month (T1) and one year (T2) after the intervention respectively. (See Fig. 1) The baseline characteristics were generally comparable between those who completed the study and those who did not (Table S1). Participants were aged 15.3 years on average (standard deviation = 1.1, range = 12–18 years). Family members of 12.5% (*n* = 125) of the students had received HPV vaccination and 13.1% (*n* = 131) had previously attended an educational talk related to HPV vaccination. Only 3.1% (*n* = 31) had prior discussions with healthcare professionals about HPV vaccination in the preceding 12 months. Detailed participant characteristics are presented in Table 1.

**Table 1:** Baseline characteristics of the student participants (*N* = 1,002)

Characteristics	Control (*n* = 517)	Intervention (*n* = 485)
Age (years) ^†^	15.3 (1.1)	15.2 (1.0)
Have received influenza vaccine		
No	154 (29.8%)	158 (32.5%)
Yes	355 (68.7%)	316 (65.2%)
Don’t know	8 (1.5%)	11 (2.3%)
Have attended a talk related to HPV vaccination		
No	447 (86.5%)	418 (86.2%)
Yes	70 (13.5%)	61 (12.6%)
Don’t know	0 (0.0%)	6 (1.2%)
Family members ever had HPV vaccination		
No	436 (84.3%)	393 (81.0%)
Yes	61 (11.8%)	64 (13.2%)
Don’t know	20 (3.9%)	28 (5.8%)
Had discussed HPV vaccination with a health professional in the past 12 months		
Haven’t seen any health professionals	272 (52.7%)	219 (45.2%)
No	223 (43.1%)	243 (50.1%)
Yes	13 (2.5%)	18 (3.7%)
Don’t know	9 (1.7%)	5 (1.0%)
Ever received information related to HPV vaccination from the following sources		
Health leaflet, brochure or poster	135 (26.1%)	132 (27.2%)
Advertisements by pharmaceutical companies	38 (7.4%)	37 (7.6%)
Health professionals	20 (3.9%)	25 (5.2%)
Family members	88 (17.0%)	105 (21.6%)
Friends, peers or colleagues	78 (15.1%)	94 (19.4%)
Schools	112 (21.7%)	101 (20.8%)
Newspaper or magazine	30 (5.8%)	36 (7.4%)
TV or radio	156 (30.2%)	159 (32.8%)
Internet	124 (24.0%)	146 (30.1%)
Social media platforms	101 (19.5%)	113 (23.3%)
Most likely to receive information related to HPV vaccination from the following sources		
Health leaflet, brochure or poster	126 (24.4%)	114 (23.5%)
Advertisements by pharmaceutical companies	19 (3.7%)	33 (6.8%)
Health professionals	130 (25.1%)	176 (36.3%)
Family members	71 (13.7%)	74 (15.3%)
Friends, peers or colleagues	52 (10.1%)	61 (12.6%)
Schools	169 (32.7%)	141 (29.1%)
Newspaper or magazine	28 (5.4%)	28 (5.8%)
TV or radio	91 (17.6%)	86 (17.7%)
Internet	112 (21.7%)	133 (27.4%)
Social media platforms	111 (21.5%)	130 (26.8%)
Data marked with^†^are presented as mean (standard deviation), all others are presented as frequency (%).		

### HPV vaccine uptake

Consistently more students in the intervention group reported receiving HPV vaccination at one month and one year after the intervention respectively than that of the control group, with 19 (4.9%) students in the intervention group versus 6 (1.3%) students in the control group at T1 (odds ratio = 1.71, 95% CI: 0.89 to 3.28), and 32 (11.9%) versus 29 (7.9%) students at T2 (odds ratio = 1.30, 95% CI: 0.50 to 3.36), though the differences between the two groups were not statistically significant. A post-hoc power analysis was conducted based on a comparison of HPV vaccination rate observed at 1-year following intervention between the two groups. The achieved power was 39.8% at 5% level of significance (2-sided).

### Intention to receive HPV vaccination

Students in the intervention group reported a greater increment in intention to receive HPV vaccination compared to students in the control group after the intervention (Group-by-time interaction B = 0.23, 95% Confidence Interval (CI), [−0.06, 0.53], *p* = 0.123).

### Attitudes towards HPV vaccination

Significantly greater changes were found in the CHIAS-C subscales of Barriers (B=−2.20, 95% CI, [−3.46, −0.94], *p* = 0.001), Risk Denial (B=−0.82, 95% CI, [−1.60, −0.05], *p* = 0.038), and Uncertainty (B = 0.72, 95% CI, [0.16, 1.27], *p* = 0.011) among the intervention group students than the control group students after the intervention, indicating that the intervention group students had more positive attitudes towards HPV vaccination. In particular, they had reduced perceptions of harms and denial of risk of HPV related infections. The increase in perceived uncertainty about HPV vaccination among the intervention group students may be attributed to their increased informational need after the intervention, which may have been the first time students were introduced to this topic.

### HPV and HPV vaccination knowledge

Students in the intervention group consistently showed improvement in their knowledge about HPV, with greater increases in the total scores of the GK23 (B = 0.94, 95% CI, [0.16, 1.72], *p* = 0.018) and the VK9 (B = 0.32, 95% CI, [−0.06, 0.70], *p* = 0.095) than that of the control group students after the intervention. Results are summarized in Table 2.

**Table 2:** Student outcome measures across the study time points

Student outcomes		Control	Intervention	B (95% CI)	*P*	Cohen’s D (95% CI) ^c^
Uptake of HPV vaccine #	T1	6 (1.3%)	19 (4.9%)	1.71 (0.89–3.28) ^a^	0.106	
	T2	29 (7.9%)	32 (11.9%)	1.30 (0.50–3.36) ^a^	0.587	
Likelihood to have HPV vaccination	T0	6.5 (2.2)	6.5 (2.2)			
[possible score range: 1 = certainly not to 10 = certainly yes]	T1	6.5 (2.1)	6.6 (2.1)	0.23 (−0.063, 0.53) ^b^	0.123	0.14 (0.00 to 0.28)
Attitudes towards HPV vaccination (CHIAS-C)						
Barriers [possible score range: 0–50]	T0	25.0 (6.9)	26.2 (7.1)			
	T1	26.4 (8.9)	25.5 (9.2)	−2.20 (−3.46, −0.94) ^b^	0.001	0.26 (0.13 to 0.40)
Harms [possible score range: 0–50]	T0	22.4 (7.2)	22.9 (7.4)			
	T1	21.8 (8.6)	22.2 (8.4)	−0.36 (−1.58, 0.86) ^b^	0.562	0.07 (−0.06 to 0.21)
Effectiveness [possible score range: 0–20]	T0	8.8 (2.4)	8.5 (2.4)			
	T1	7.6 (3.4)	7.8 (3.3)	0.42 (−0.05, 0.89) ^b^	0.082	0.10 (−0.03 to 0.24)
Risk denial [possible score range: 0–30]	T0	12.3 (4.8)	12.9 (5.1)			
	T1	12.2 (5.3)	12.1 (5.1)	−0.82 (−1.60, −0.05) ^b^	0.038	0.16 (0.03 to 0.30)
Uncertainty [possible score range: 0–20]	T0	11.0 (3.1)	10.2 (3.3)			
	T1	11.2 (3.9)	11.2 (3.7)	0.72 (0.16, 1.27) ^b^	0.011	0.19 (0.05 to 0.33)
HPV knowledge scale (GK23)						
Total score [possible score range: 0–23]	T0	6.9 (5.3)	6.9 (5.1)			
	T1	7.8 (6.0)	8.7 (6.0)	0.94 (1.62, 1.72) ^b^	0.018	0.17 (0.03 to 0.30)
Vaccination knowledge scale (VK9)						
Total score [possible score range: 0–9]	T0	2.9 (2.5)	3.0 (2.4)			
	T1	3.3 (2.7)	3.7 (2.9)	0.32 (−0.06, 0.70) ^b^	0.095	0.09 (−0.04 to 0.23)

Data marked with # are presented as frequency (%), all others are presented as mean (standard deviation)

CHIAS-C: The Chinese version of the modified Carolina Human papillomavirus Immunisation Attitudes and Beliefs Scale (Adolescent version). GK23: The Chinese version of the 23-item HPV knowledge scale. VK9: The Chinese version of the 9-item vaccination knowledge scale. B: regression coefficient estimated by using up to three-level random intercept mixed-effects models accounting for intra-correlated clustered and repeated measures data

^a^ Odds ratio (95% CI) of HPV vaccine uptake

^b^ The group-by-time interaction term

^c^ Cohen’s d effect sizes were calculated based on the change score at T1 with respect to T0

## Discussion

Health education is an effective method of addressing vaccine hesitancy and promoting HPV vaccination, though effects on actual vaccine uptake vary [29, 30]. Our study suggests that the MDL-SHPVP may be beneficial in improving adolescent female students’ knowledge and attitudes towards HPV vaccination, demonstrating value in providing students with reliable and relevant information which may empower them to make informed health decisions and receive HPV vaccination. However, the effect sizes are conventionally regarded as small (Table 2). While improvements in the outcomes of vaccine uptake and intention were not significant, the higher vaccination rate in the intervention group indicates that the programme could have some impact in prompting adolescents to receive the vaccination. Notably, participants in the intervention group demonstrated 30% higher odds of receiving the vaccination 1-year after intervention compared to their counterparts in the control group. Owing to the COVID-19 pandemic, the final sample size was smaller than planned and the actual power achieved for the primary outcome of HPV vaccination rate at 1-year after intervention was less than 40% at 5% level of significance (2-sided), which consequently reduced the chance of detecting the statistical significance of the outcomes between groups.

As a school-based educational intervention, the programme positively impacted students’ knowledge, attitudes, and intention to receive HPV vaccination. Notably, attitudinal improvements were found in the subscales of “Barriers” and “Risk denial”, suggesting that the educational content helped students perceive less harm in receiving the vaccination and better understand their risk status. Since previous studies have noted that low perceived risk of HPV infection is a common reason for refusing HPV vaccination [8, 31], adolescents’ heightened understanding of their susceptibility to infection combined with stronger awareness about the vaccination is an encouraging finding that could improve uptake. On the other hand, higher uncertainty regarding vaccination was also reported, alluding to adolescents’ complex informational needs. It is likely that despite increasing adolescents’ understanding of HPV vaccination, the intervention may have prompted further questions and doubts since it was their first time learning about this topic. As the programme was delivered in a one-off session, students who did not clarify their concerns during the intervention may have had questions remaining and thus reported higher uncertainty afterwards. To address this issue, multi-session interventions should be considered in order to provide students with more opportunities to discuss their doubts and raise their confidence in receiving HPV vaccination. According to a study on school-based HPV vaccination promotion campaigns in the United States, schools that arranged a variety of intervention activities for longer durations (such as organizing HPV vaccination awareness weeks) reported higher vaccine uptake, indicating that intervention length is an important factor in reinforcing awareness of HPV vaccination [32]. 

In terms of vaccine uptake, more students in the intervention group were vaccinated one year post-intervention compared to those in the control group, indicating a positive effect on the vaccine uptake rate. However, statistical significance was not reached and the vaccination rate was suboptimal, indicating that programmes focused on education alone may not be enough to boost vaccine uptake and that strategies specifically encouraging students to seek and receive the vaccination should be incorporated. For example, a systematic review and meta-analysis of 34 studies comparing educational and digital reminder-based HPV vaccine promotion interventions found that combining education with reminder-based strategies was more effective in raising HPV vaccination rates compared to education-focused programmes which may fall short in inducing participants to initiate HPV vaccination [33]. In the current study, while there are several factors which may have led to a low vaccination uptake rate, it is likely that the simultaneous occurrence of the COVID-19 pandemic was a significant reason, with refusal or delays in receiving the HPV vaccine driven by vaccine fatigue and concerns regarding potential contraindications with COVID-19 vaccines. This is consistent with global trends of reduced HPV vaccination rates associated with COVID-19-related delays, with factors such as limited healthcare access, reduced prioritization of HPV vaccination, and logistical challenges affecting HPV vaccine initiation as well as completion [34–36]. 

Similarly, a non-significant increase in vaccine intention was also observed in the intervention group, suggesting that although the intervention successfully communicated the necessity of receiving HPV immunization, certain barriers to the vaccination still persisted. Indeed, a systematic review on educational interventions for adolescents has suggested that intention can be an unreliable indicator for vaccine uptake, though gaps in the literature remain and more studies are required to better understand the relationship between intention and actual vaccine uptake [37]. There are several reasons why higher vaccine intention among adolescents may not necessarily translate into higher vaccine uptake, particularly as they largely depend on parents for health-related decision-making, with parental knowledge, attitudes, and behaviors such as seeking information about HPV vaccination affecting vaccination outcomes [38]. Moreover, as the vaccination would be an out-of-pocket expense for girls not covered by subsidized immunization programmes, the cost of receiving vaccination may also be a significant barrier, particularly for lower- to middle-income households who may not consider the vaccination to be an urgent priority [31]. Involving parents in educational interventions to address their concerns could thus potentially encourage the conversion of vaccination intent into uptake.

Along with the recent introduction of the government-subsidized HPV vaccination scheme for female students in Grades 5–6 in Hong Kong, the intervention could play an important role in strengthening students’ knowledge of HPV vaccination and cultivating positive attitudes towards immunization, thus enabling them to make an informed health decision. Especially given the notably low percentage (13.1%) of students who had attended HPV vaccination talks previously, there is an evident need for a timely introduction to this topic and the MDL-SHPVP could present as an effective option. The educational materials in the programme could also be adapted to the health needs of adolescent boys and evaluated in RCTs for their impact on HPV vaccine acceptance and uptake in order to boost wider vaccine coverage. Moreover, given the significant role of parents in HPV vaccine initiation for adolescents, future studies could investigate interventions with more active parent/guardian participation and engagement to better understand their effect on HPV vaccine uptake.

### Limitations

This study has some limitations. Due to the social distancing guidelines during the COVID-19 pandemic, intervention sessions were mainly delivered either in a hybrid face-to-face or online video conferencing format, with data collection similarly conducted either through physical or online questionnaires. The difference in methods of intervention delivery may have impacted the effectiveness of the intervention and also contributed to the loss of participants during follow-up data collection. Moreover, non-significant results, particularly the vaccine uptake, were likely a consequence of the reduced small sample size and hence statistical power. It should be noted that the actual power for the primary outcome ‘HPV vaccine uptake rate’ was less than 40%. A larger-scale study involving a bigger sample and more consistent format of intervention delivery and data collection is therefore warranted to retain participants and provide additional insights into the effects of school-based health promotion programmes for HPV vaccination among female adolescents.

## Conclusion

The MDL-SHPVP programme was evaluated in this cluster RCT, with beneficial effects observed in students’ knowledge and attitudes toward HPV vaccination. The MDL-SHPVP has the potential to play a crucial role in empowering adolescent girls to make informed decisions about HPV vaccination by addressing gaps in health knowledge and promoting favourable attitudes. Though a non-significantly greater vaccine uptake rate was observed in the intervention group, additional strategies to prompt initiation of HPV vaccination could be incorporated. Future research may explore the integration of different components and a lengthier intervention involving other stakeholders such as school staff, healthcare providers, and parents to better address informational needs and overcome barriers to HPV vaccination. The programme content can also be adapted to meet the needs of other demographics such as older girls and adolescent boys, and further evaluated to determine its impact on their HPV vaccine uptake, knowledge, and attitudes.

## Supplementary Information

Supplementary Material 1.

Supplementary Material 2.

## Data Availability

The data that supports the findings of this study are available from the corresponding author (JPCC) upon reasonable request.

## Abbreviations

CHIAS-C: The Chinese version of the modified Carolina Human papillomavirus Immunisation Attitudes and Beliefs Scale (Adolescent version)
GK23: HPV knowledge scale
HPV: Human papillomavirus
MDL-SHPVP: Multidisciplinary team-led school-based HPV vaccination health-promotion programme
CRT: Cluster randomized controlled trial
VK9: Vaccination knowledge scale
