# Supplementary material for: Effects of an educational health promotion intervention to improve human papillomavirus vaccination acceptance and uptake among adolescent girls: a cluster randomized controlled trial
Source: BMC Public Health. 2025 Oct 9;25:3419. doi: 10.1186/s12889-025-24511-4 (PMC12513138; doi:10.1186/s12889-025-24511-4)
Supplement: Supplementary file 1 — Supplementary Material 1. [file 12889_2025_24511_MOESM1_ESM.docx]

**Supplementary File 1 – Study Questionnaire (HPV vaccine uptake and sociodemographic sections)**

**Immunisation status**

1. Have you ever received HPV vaccination?

|  | Yes |
| --- | --- |
|  | No |

1. Where have you received HPV vaccination?

Instructions: Select only one

|  | Public health clinic |
| --- | --- |
|  | Private clinic |
|  | Hospital |
|  | School |
|  | If other, please specify: ________________ |

1. How old were you when you received HPV vaccination?

At _______ year(s) old

**Sociodemographic Survey and Previous vaccinations**

**Demographics**

1. Age: __________

**Immunisation history**

1. Have you ever received any vaccine for influenza?

|  | Yes |
| --- | --- |
|  | No |

1. Have you ever received any vaccine for HPV?

|  | Yes |
| --- | --- |
|  | No |
