# Supplementary material for: Effects of an educational health promotion intervention to improve human papillomavirus vaccination acceptance and uptake among adolescent girls: a cluster randomized controlled trial
Source: BMC Public Health. 2025 Oct 9;25:3419. doi: 10.1186/s12889-025-24511-4 (PMC12513138; doi:10.1186/s12889-025-24511-4)
Supplement: Supplementary file 2 — Supplementary Material 2. [file 12889_2025_24511_MOESM2_ESM.docx]

Table S1. Baseline characteristics between those who completed the study and those who did not complete the study

| ***Characteristics*** | All  (n=1002) | Dropouts  (n=364) | Completers (n=638) |
| --- | --- | --- | --- |
| Age (years) ^†^ | 15.3 (1.1) | 15.4 (1.1) | 15.2 (1.0) |
| Have received influenza vaccine |  |  |  |
| No | 312 (31.1%) | 104 (28.6%) | 208 (32.6%) |
| Yes | 671 (67.0%) | 254 (69.8%) | 417 (65.4%) |
| Don’t know | 19 (1.9%) | 6 (1.6%) | 13 (2.0%) |
| Have attended a talk related to HPV vaccination |  |  |  |
| No | 865 (86.3%) | 322 (88.5%) | 543 (85.1%) |
| Yes | 131 (13.1%) | 40 (11.0%) | 91 (14.3%) |
| Don’t know | 6 (0.6%) | 2 (0.5%) | 4 (0.6%) |
| Family members ever had HPV vaccination |  |  |  |
| No | 829 (82.7%) | 322 (88.5%) | 507 (79.5%) |
| Yes | 125 (12.5%) | 31 (8.5%) | 94 (14.7%) |
| Don’t know | 48 (4.8%) | 11 (3.0%) | 37 (5.8%) |
| Had discussed HPV vaccination with a health professional in the past 12 months |  |  |  |
| Haven’t seen any health professionals | 491 (49.0%) | 154 (42.4%) | 337 (52.8%) |
| No | 466 (46.5%) | 201 (55.2%) | 265 (41.6%) |
| Yes | 31 (3.1%) | 6 (1.6%) | 25 (3.9%) |
| Don’t know | 14 (1.4%) | 3 (0.8%) | 11 (1.7%) |

Data marked with ^†^ are presented as mean (standard deviation), all others are presented as frequency (%).
